# Prostate-specific antigen in the cerebrospinal fluid leads to diagnosis of solitary cauda equina metastasis: a unique case report and review of the literature.

**DOI:** 10.1038/bjc.1998.397

**Published:** 1998-06

**Authors:** B. Schaller, A. Merlo, E. Kirsch, K. Lehmann, P. R. Huber, P. Lyrer, A. J. Steck, O. Gratzl

**Affiliations:** Department of Neurological Surgery, University Hospitals, Basle, Switzerland.

## Abstract

**Images:**


British Journal of Cancer (1998) 77(12), 2386–2389
© 1998 Cancer Research Campaign

### Prostate-specific antigen in the cerebrospinal fluid leads to diagnosis of solitary cauda equina metastasis: a unique case report and review of the literature

**B Schaller[1], A Merlo[1], E Kirsch[2], K Lehmann[3], PR Huber[4], P Lyrer[5], AJ Steck[5] and O Gratzl[1]**

Departments of [1]Neurological Surgery, [2]Neuroradiology and [3]Urology, [4]Central Laboratories and [5]Department of Neurology, University Hospitals, Spitalstrasse 21, CH-4031 Basle, Switzerland

**Summary** A 79-year-old male patient presented with a subacute cauda syndrome caused by an intradural metastasis of the lumbosacral caudate fibres from an adenocarcinoma of the prostate, which had been treated 5 years earlier with external beam radiation therapy. Diagnosis could not be established by repeated magnetic resonance images (MRIs) during a 2-year period of increasingly severe radicular pain. Eventually, a small tumour mass could be visualized on the fourth MRI. Repeated normal serum prostate-specific antigen (PSA) did not hint at a prostate cancer metastasis (range 2.4–5.1 ng ml$^{-1}$); however, PSA in the cerebrospinal fluid was found to be elevated (29.1 ng ml$^{-1}$). Empirical radiation therapy of the caudate region did not improve radicular pain. Therefore, an exploratory surgical procedure was conducted, which confirmed the suspicion of an intradural prostate cancer metastasis. In conclusion, PSA in the cerebrospinal fluid provides a useful diagnostic tool for detecting intradural prostate cancer metastasis.

**Keywords:** intradural spinal metastasis; caudate fibres; prostate-specific antigen; prostate cancer; alpha-1-anti-chymotrypsin

Clinically occult carcinoma of the prostate has been found at autopsy series in around 70% of men over the age of 80 (Grant et al, 1994; Oschmann et al, 1994). In manifest disease with metastatic spread, skeletal, pulmonary as well as hepatic metastases predominate (Elkin and Mueller, 1954), but intradurally located metastases of the spinal axis are uncommon. Since 1950, 59 patients with histologically confirmed intradural spinal metastasis have been found in scattered reports (Perrin et al, 1982; Chow and McCutcheon, 1996), containing only one case of prostate cancer. It is, however, generally accepted that the blood–brain barrier has to be disrupted in intradural carcinomatosis (Siegal et al, 1987), and therefore that haematogenous dissemination from the primary tumour as well as spinal or epidural metastases by embolization may be the most important and common mechanism for spread of tumour cells into the spinal subrachnoid space (Perrin et al, 1982; Chow and McCutcheon, 1996).

### CASE REPORT

A 79-year old man was evaluated for a 3-year history of increasingly severe radicular pain projecting into the left lumbosacral dermatomes. His medical history included a prostate cancer (T3, N0, M0) diagnosed 5 years earlier by a needle biopsy (moderately differentiated adenocarcinoma of the prostate) and treated with fractionated external beam radiation therapy (45 MeV, 6000 cGy cumulative). Two years later, a radicular pain syndrome developed insidiously, projecting into the left-sided lumbosacral dermatomes L5–S3 and accompanied by a diminished left side motor function (abductor muscle BMA grade 4, iliopsoas muscle grade 4–5, quadriceps muscle grade 4–5, gastrocnemius muscle grade 0–1, peroneus muscle grade 0–1, extensor hallucis longus and digitorum brevis muscles grade 0–1) as a pronounced hypalgesia in the left dermatomes L4–S1. There was urinary and bowel incontinence. Because radicular pain intensified, clinical and laboratory tests [prostate-specific antigen (PSA), prostate-specific acid phosphatase (PAP), cerebro-spinal fluid (CSF) cytology, CSF infection] were repeated every 6 months over the next 2 years with no pathological findings. Radiological investigations using abdominal ultrasound, excretory urography, skeletal scintigraphy and abdominal computerized tomography (CT) could not detect metastatic disease; even repeated myelographic and magnetic resonance imaging (MRI) studies did not show signs of lumbosacral metastasis (Figure 1). The serum PSA level too was found to be within normal range, but steadily increasing in the range 2.4–5.1 ng ml$^{-1}$ (Figure 2). A lumbar puncture showed a PSA in the CSF of 29 ng ml$^{-1}$ [a main part of free PSA and a minor part of PSA complexed with alpha-1-anti-chymotrypsin (ACT)] and serum PSA was 5.1 ng ml$^{-1}$ (a minor part of free PSA and a main part of PSA complexed with ACT). The method used to measure PSA concentration has been described previously in detail (Huber et al, 1995). The fourth MRI of the lumbar spine showed an intradural, hypointense lesion likely to be a metastasis of the cauda fibre between L5 and S3 (Figure 3) on T2-weighted and T1-weighted images, clearly enhanced after administration of gadolinium-Dota (Dotarem Guerbet Laboratoires, Paris, France). The pronounced pain syndrome was treated empirically with external beam radiation therapy of the spine (25 MeV), which had to be interrupted after a total dose of 32 Gy because of progression and cast doubt on the metastatic nature of the lesion. Therefore, an explorative laminectomy L5–S3 was conducted. After opening the dural sac, caudate fibres were found to be surrounded by hard

*Received 10 October 1997*
*Revised 17 November 1997*
*Accepted 26 November 1997*

*Correspondence to:* B Schaller, Neurosurgical Clinic, Department of Surgery, University Hospital, Spitalstrasse 21, CH-4031 Basle, Switzerland

**Figure 1** T1-weighted magnetic resonance images in sagittal plane before (**A**) and axial plane after (**B**) contrast administration. No evidence of tumour in the lumbosacral spinal canal

**Figure 2** Line chart showing the course of PSA during illness. PSA in serum under 10 ng ml$^{-1}$ is indicated as normal value. Time 0 means the beginning of radiation therapy of the prostate. □, PSA (serum); △, PSA (CSF)

fibrous tissue that was partially removed. Histological examination revealed a poorly differentiated carcinoma of the prostate (Gleason grade 4; Gleason, 1966). The post-operative course was uneventful.

Eighteen months after surgical exploration, the patient's condition showed progressive neurological detoriation. Gonadotropin-releasing hormone analogues had been administered for 3 months without effect. Orchiectomy was declined by the patient.

### DISCUSSION

We present a unique case of an intradural metastasis from prostate cancer in which the exclusive elevation of cerebrospinal fluid PSA was instrumental in establishing the diagnosis. In addition, this is the second case report of a histologically proven intradural extramedullary spinal metastasis originating from prostate carcinoma since 1959.

The criteria for leptomeningeal carcinomatosis are not uniform, but can usually be established when two or more anatomical regions of the central nervous system are involved (Olson et al, 1974). It is often misinterpreted as chemotherapy- or radiotherapy-induced polyneuropathy or meningoradiculitis (Schuknecht et al, 1982), which may cause a substantial delay in definite diagnosis. Gadolinium (Gd)-enhanced MR imaging has been shown to be superior to myelography and to CT/myelography in localizing intradural metastatic lesions (Sze et al, 1988). But even with the most sensitive technique – Gd-enhanced MR imaging – one-third of cases with proven meningeal carcinomatosis were missed (Sze et al, 1989), or diagnosis was delayed as in our case. Probably because high-signal CSF in T2-weighted sequences renders high-signal subarachnoid lesions inperceptible and accounts for its low sensitivity and the limited value of T2-weighted images (Schuknecht et al, 1982).

The finding of malignant cells in the CSF cytology is difficult, as several lumbar punctures might be required. The false-negative rate ranges from 27% to 90% (Elkin and Mueller, 1954; Oschmann et al, 1994), more often in cases of focal than disseminated disease (Chow and McCutcheon, 1996). An important feature in this patient was the elevated PSA in the CSF with low

© Cancer Research Campaign 1998

**Figure 3** Control magnetic resonance examination 2 years after onset of symptoms. Sagittal T2-weighted images (**A**) show a spindle-like lesion intradural on the level of L5–S3 (arrow). Axial T1-weighted image (**B**) before contrast administration shows a slightly hyperintense tumour in comparison with CSF, with almost total occlusion of the dural sack (arrowheads). On saggital gadolinium enhanced T1-weighted images (**C**) the tumour shows a homogeneous enhancement with intermediate signal intensity (curved arrow)

*© Cancer Research Campaign 1998*

PSA levels in the serum. ACT, one of the two major proteinase inhibitors of active PSA zymogen, is present in serum as well as in the CSF, in the former in higher concentrations (Lilja et al, 1992; Huber et al, 1995; Oishi et al, 1996). Because the complex formation of PSA with its inhibitor ACT is stable, the elevated concentrations of complex formation of PSA with its inhibitor ACT in the CSF as compared with the serum proves a real intrathecal secretion of PSA (Lilja et al, 1992). The small volume of the intradural spinal metastasis may not have produced enough PSA in the CSF to pass the moderately altered blood–CSF barrier (albumin quotient 23.0, IgG index 6.4) and build up a serum PSA level above threshold. This clearly shows that assessment of the PSA level in the CSF is a useful diagnostic tool for detecting intradural prostate cancer metastasis. It is not clear whether the slight increase in serum PSA signifies recurrence or variations within normal range (Figure 1).

Although patients with untreated leptomeningeal carcinomatosis generally die within 4–8 weeks from the time of diagnosis (Olson et al, 1974), the rare cases of focal intradural metastatic disease may be approached surgically to establish diagnosis or to excise the lesion (Chow and McCutcheon, 1996). However, Chow and McCutcheon (1996) presented two of ten cases of focal meningeal carcinomatosis (alveolar soft-part sarcoma, poorly differentiated adenocarcinoma with no known primary tumour) with a post-operative survival of more than 1 year after surgery. In the special case of established metastatic prostate cancer, anti-androgen therapy is primary treatment that may be supplemented with regional radiation therapy.

### CONCLUSION

Histologically confirmed intradural spinal metastasis is a rare event. Even with the most sensitive techniques, the diagnosis of such a lesion is difficult and often delayed by several months or years. The assessment of PSA in the CSF can be useful in establishing the diagnosis of spinal intradural metastasis from prostate cancer, even if serum PSA levels are normal.

### REFERENCES

Chow TSF and McCutcheon IE (1996) The surgical treatment of metastatic spinal tumors within the intradural extramedullary compartment. *J Neurosurg* **85**: 225–230

Elkin M and Mueller HP (1954) Metastases from cancer of the prostate. Autopsy and roentgenological findings. *Cancer* **7**: 1246–1248

Gleason DF (1966) Classification of prostatic carcinomas. *Cancer Chemother Rep* **50**: 125

Grant R, Naylor B, Greenberg HS and Junck L (1994) Clinical outcome in aggressively treated meningeal carcinomatosis. *Arch Neurol* **51**: 457–461

Huber PR and Mattarelli G, Strittmatter B, van Steenbrugge GJ, Schmid HP, Maurer A (1995) In vivo and in vitro complex formation of prostate specific antigen with alpha-1-anti-chymotrypsin. *Prostate* **27**: 166–175

Lilja H, Cockett ATK and Abrahamson PA (1992) Prostate specific antigen predominantly forms a complex with alpha-1-anti-chymotrypsin in blood. *Cancer* **70**: 230–234

Oishi M, Mochizuki Y, Yoshihashi H, Takasu T and Nakano E (1996) Laboratory examination correlated with severity of dementia. *Ann Clin Lab Sci* **26**: 340–345

Olson ME, Chernik NL and Posner JB (1974) Infiltration of the leptomeninges by systemic cancer. A clinical and pathologic study. *Arch Neurol* **30**: 122–137

Oschmann P, Keeps M, Völker J and Dorndorf W (1994) Meningeal carcinomatosis: CSF cytology, immunochemistry and biochemical tumor markers. *Acta Neurol Scand* **89**: 395–399

Perrin RG, Livingston KE and Aarabi B (1982) Intradural extramedullary spinal metastasis. A report of 10 cases. *J Neurosurg* **56**: 835–837

Schuknecht B, Huber P, Büller B and Nadjmi M (1992) Spinal leptomeningeal neoplastic disease. *Eur Neurol* **32**: 11–16

Siegal T, Sandbank U, Gabizon A, Siegal T, Mizrachi R, Ben-Davis E and Catane R (1987) Alteration in blood CSF barrier in experimental meningeal carcinomatosis. *J Neurooncol* **4**: 233–242

Sze G, Abramson A, Krol G, Liu D, Amster J, Zimmermann RD and Reck MDF (1988) Gadolinium-DTPA in the evaluation of intradural extramedullary spinal disease. *Am J Radiol* **150**: 911–921

Sze G, Soletsky S, Bronen R and Krol G (1989) MR imaging of the cranial meninges with emphasis on contrast enhancement and meningeal carcinomatosis. *Am J Radiol* **10**: 965–975

© Cancer Research Campaign 1998


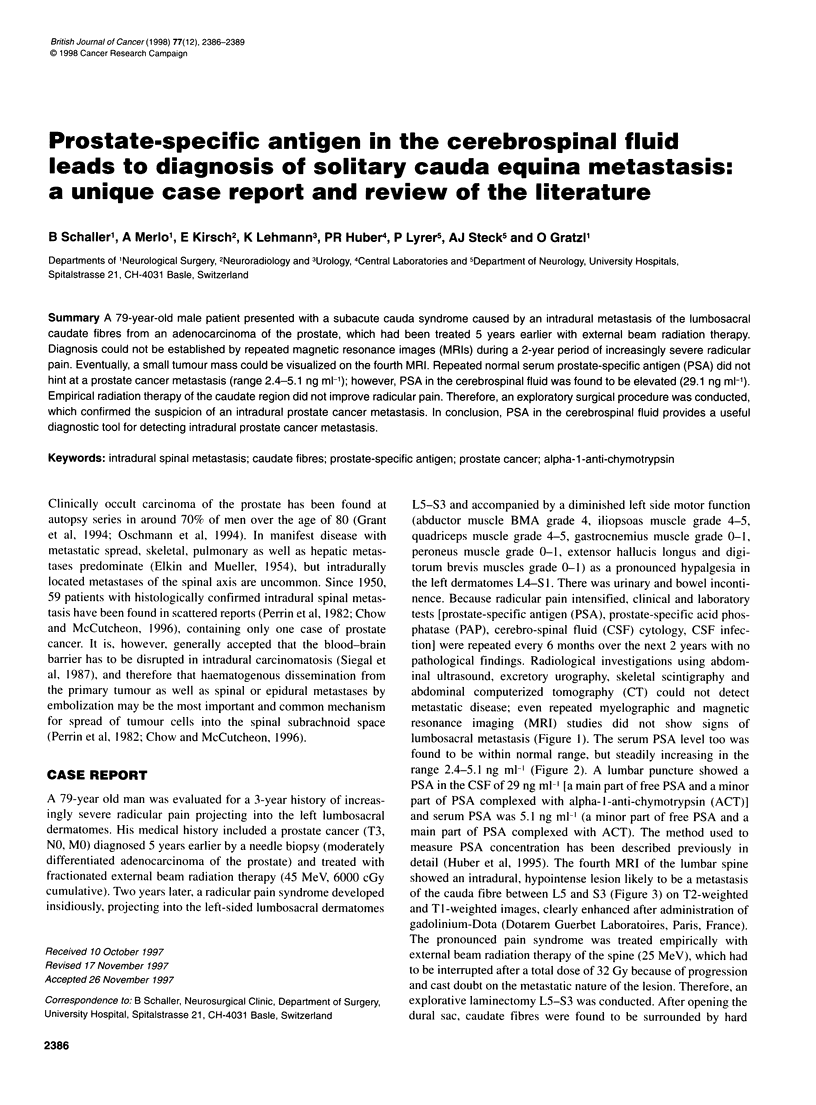

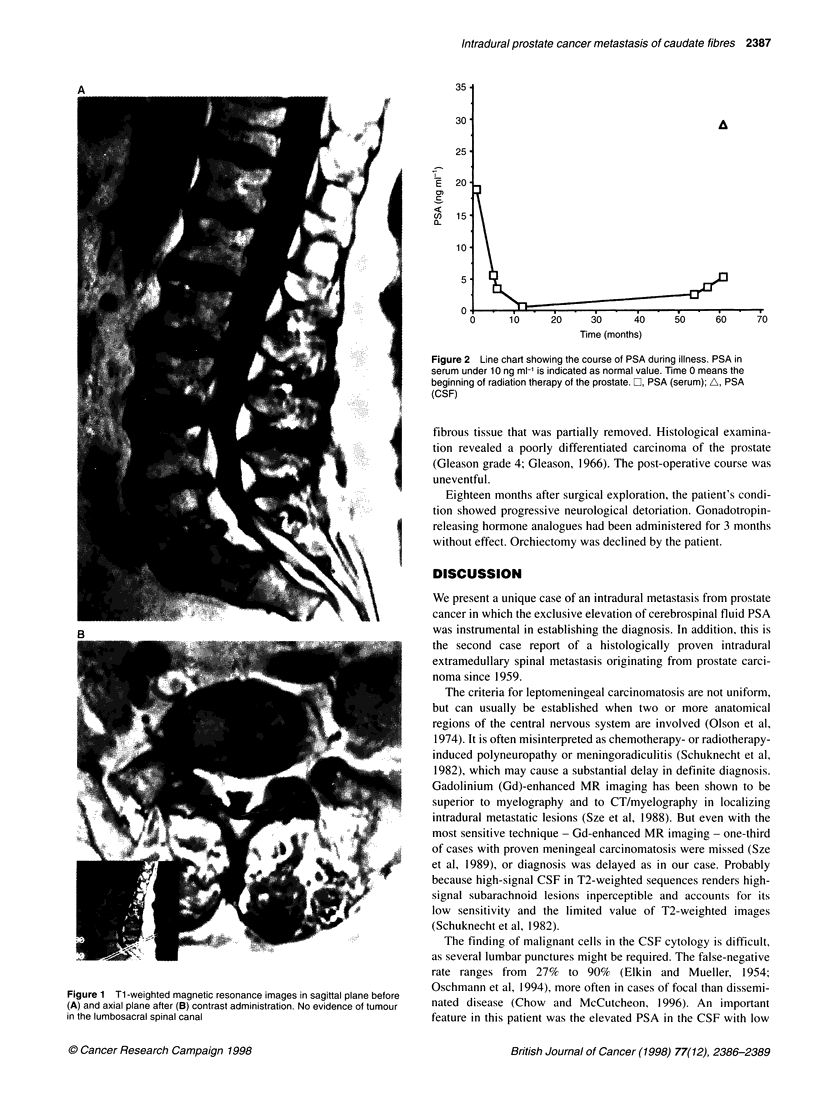

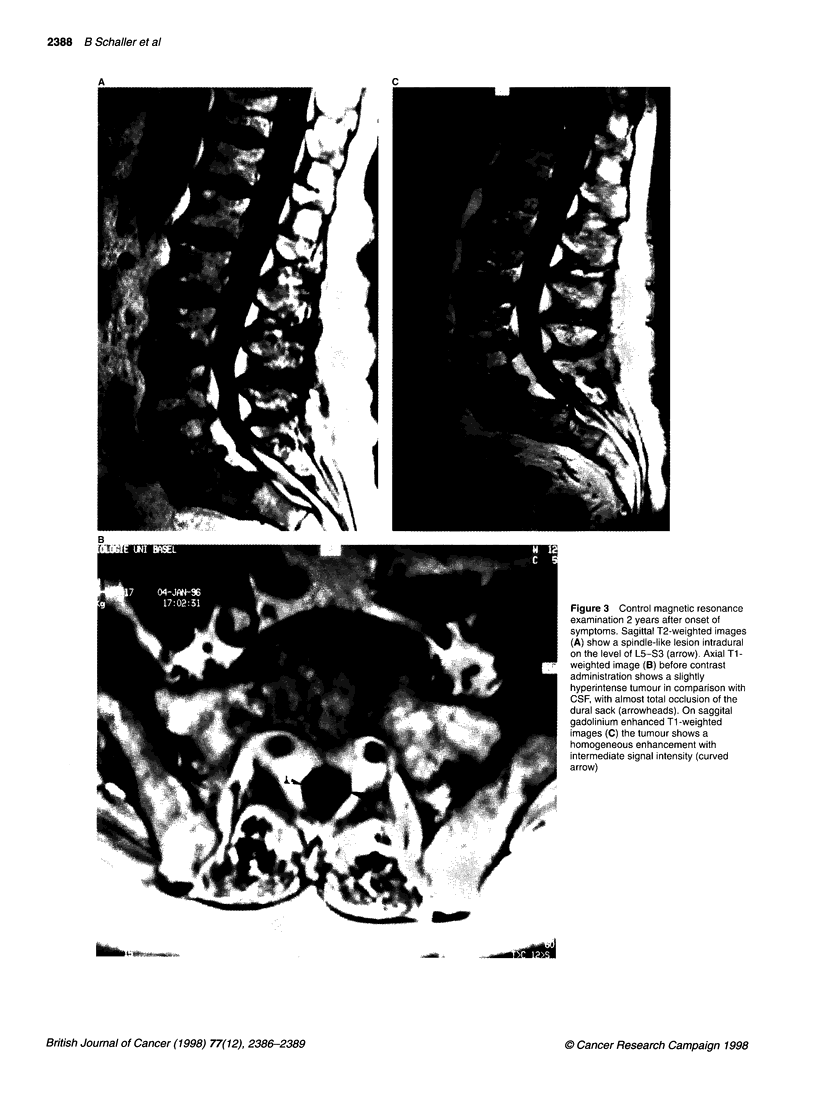

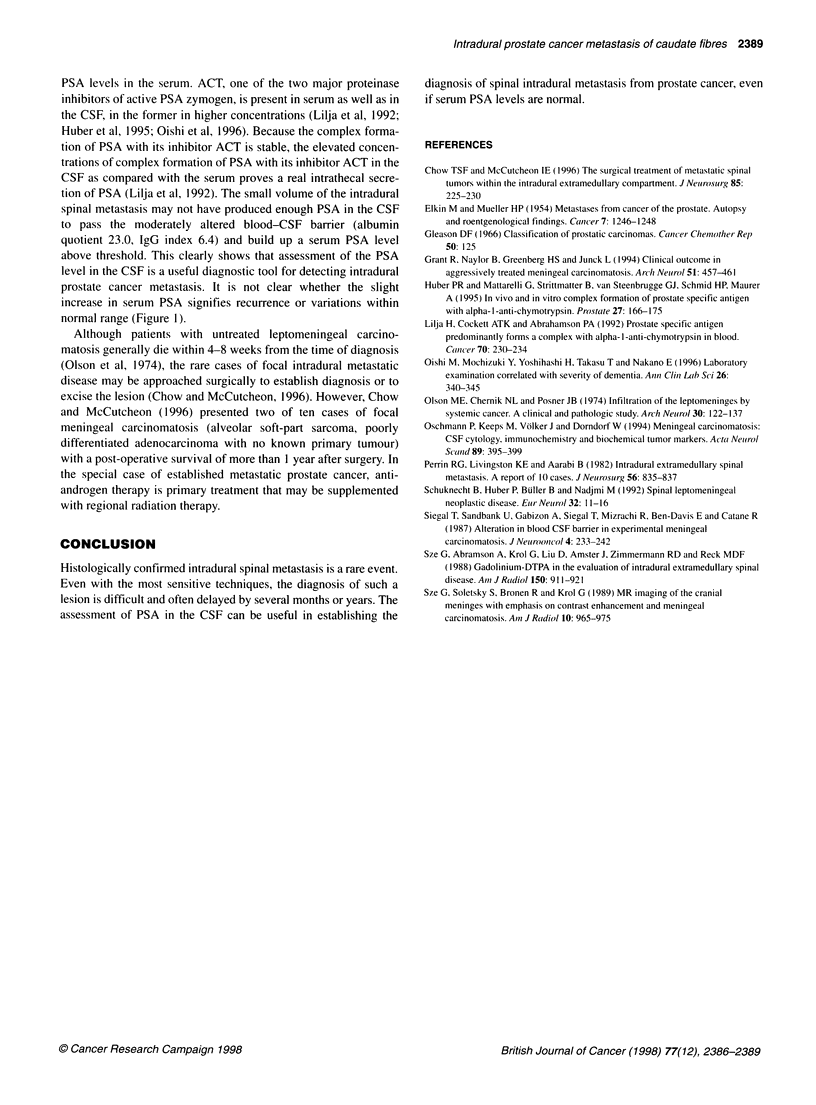


## References

[OCR_00249] Chow T. S., McCutcheon I. E. (1996). The surgical treatment of metastatic spinal tumors within the intradural extramedullary compartment.. J Neurosurg.

[OCR_00254] ELKIN M., MUELLER H. P. (1954). Metastases from cancer of the prostate; autopsy and roentgenological findings.. Cancer.

[OCR_00258] Gleason D. F. (1966). Classification of prostatic carcinomas.. Cancer Chemother Rep.

[OCR_00262] Grant R., Naylor B., Greenberg H. S., Junck L. (1994). Clinical outcome in aggressively treated meningeal carcinomatosis.. Arch Neurol.

[OCR_00266] Huber P. R., Mattarelli G., Strittmatter B., van Steenbrugge G. J., Schmid H. P., Maurer A. (1995). In vivo and in vitro complex formation of prostate specific antigen with alpha 1-anti-chymotrypsin.. Prostate.

[OCR_00271] Lilja H., Cockett A. T., Abrahamsson P. A. (1992). Prostate specific antigen predominantly forms a complex with alpha 1-antichymotrypsin in blood. Implications for procedures to measure prostate specific antigen in serum.. Cancer.

[OCR_00276] Oishi M., Mochizuki Y., Yoshihashi H., Takasu T., Nakano E. (1996). Laboratory examinations correlated with severity of dementia.. Ann Clin Lab Sci.

[OCR_00281] Olson M. E., Chernik N. L., Posner J. B. (1974). Infiltration of the leptomeninges by systemic cancer. A clinical and pathologic study.. Arch Neurol.

[OCR_00285] Oschmann P., Kaps M., Völker J., Dorndorf W. (1994). Meningeal carcinomatosis: CSF cytology, immunocytochemistry and biochemical tumor markers.. Acta Neurol Scand.

[OCR_00298] Siegal T., Sandbank U., Gabizon A., Siegal T., Mizrachi R., Ben-David E., Catane R. (1987). Alteration of blood-brain-CSF barrier in experimental meningeal carcinomatosis. A morphologic and adriamycin-penetration study.. J Neurooncol.

[OCR_00303] Sze G., Abramson A., Krol G., Liu D., Amster J., Zimmerman R. D., Deck M. D. (1988). Gadolinium-DTPA in the evaluation of intradural extramedullary spinal disease.. AJR Am J Roentgenol.

[OCR_00308] Sze G., Soletsky S., Bronen R., Krol G. (1989). MR imaging of the cranial meninges with emphasis on contrast enhancement and meningeal carcinomatosis.. AJNR Am J Neuroradiol.

